# Vulnerability and Protective Factors for PTSD and Depression Symptoms Among Healthcare Workers During COVID-19: A Machine Learning Approach

**DOI:** 10.3389/fpsyt.2021.752870

**Published:** 2022-01-12

**Authors:** Liana C. L. Portugal, Camila Monteiro Fabricio Gama, Raquel Menezes Gonçalves, Mauro Vitor Mendlowicz, Fátima Smith Erthal, Izabela Mocaiber, Konstantinos Tsirlis, Eliane Volchan, Isabel Antunes David, Mirtes Garcia Pereira, Leticia de Oliveira

**Affiliations:** ^1^Neurophysiology Laboratory, Department of Physiological Sciences, Roberto Alcantara Gomes Biology Institute, Biomedical Center, State University of Rio de Janeiro, Rio de Janeiro, Brazil; ^2^Laboratory of Neurophysiology of Behavior, Department of Physiology and Pharmacology, Biomedical Institute, Federal Fluminense University, Rio de Janeiro, Brazil; ^3^Department of Psychiatry and Mental Health, Fluminense Federal University, Rio de Janeiro, Brazil; ^4^Laboratory of Neurobiology, Institute of Biophysics Carlos Chagas Filho, Rio de Janeiro, Brazil; ^5^Laboratory of Cognitive Psychophysiology, Department of Natural Sciences, Institute of Humanities and Health, Federal Fluminense University, Rio de Janeiro, Brazil; ^6^Centre for Medical Image Computing, University College London, London, United Kingdom

**Keywords:** COVID-19, PTSD, depression, healthcare worker (HCW), machine learning

## Abstract

**Background:** Healthcare workers are at high risk for developing mental health problems during the COVID-19 pandemic. There is an urgent need to identify vulnerability and protective factors related to the severity of psychiatric symptoms among healthcare workers to implement targeted prevention and intervention programs to reduce the mental health burden worldwide during COVID-19.

**Objective:** The present study aimed to apply a machine learning approach to predict depression and PTSD symptoms based on psychometric questions that assessed: (1) the level of stress due to being isolated from one's family; (2) professional recognition before and during the pandemic; and (3) altruistic acceptance of risk during the COVID-19 pandemic among healthcare workers.

**Methods:** A total of 437 healthcare workers who experienced some level of isolation at the time of the pandemic participated in the study. Data were collected using a web survey conducted between June 12, 2020, and September 19, 2020. We trained two regression models to predict PTSD and depression symptoms. Pattern regression analyses consisted of a linear epsilon-insensitive support vector machine (ε-SVM). Predicted and actual clinical scores were compared using Pearson's correlation coefficient (r), the coefficient of determination (r^2^), and the normalized mean squared error (NMSE) to evaluate the model performance. A permutation test was applied to estimate significance levels.

**Results:** Results were significant using two different cross-validation strategies to significantly decode both PTSD and depression symptoms. For all of the models, the stress due to social isolation and professional recognition were the variables with the greatest contributions to the predictive function. Interestingly, professional recognition had a negative predictive value, indicating an inverse relationship with PTSD and depression symptoms.

**Conclusions:** Our findings emphasize the protective role of professional recognition and the vulnerability role of the level of stress due to social isolation in the severity of posttraumatic stress and depression symptoms. The insights gleaned from the current study will advance efforts in terms of intervention programs and public health messaging.

## Introduction

Coronavirus disease 2019 (COVID-19) is an infectious disease caused by the novel coronavirus (SARS-Cov2). In March 2020, the World Health Organization (WHO) characterized COVID-19 as a pandemic due to the rapid increase in the number of cases, putting the planet in a state of maximum alert (1). Driven by an infectious new variant, a lack of containment measures and a patchy vaccine rollout, Brazil has become the epicenter of the COVID-19 pandemic. According to the most recent WHO estimates, Brazil has the highest numbers of new deaths in the Americas (2). During the period of our research, Brazil surpassed 4.5 million COVID-19 cases, and more than 136,000 Brazilians have died from COVID-19 since the start of the pandemic (3). At the time of the research, an effective vaccine or medicine was not available to address COVID-19, and the most efficient strategies for controlling the COVID-19 pandemic were preventive measures and social distancing. According to an article in Lancet, Brazil was considered to have had one of the worst responses to the pandemic internationally and to have committed numerous governmental mistakes (4).

In this context, the COVID-19 pandemic not only raises physical health concerns for the entire population but also has consequences on the mental health of individuals in both the short and long terms (5–8), particularly among healthcare workers, a group with higher risks of infection and of transmitting the disease to their families and coworkers (9, 10). In fact, studies from previous epidemics, such as SARS, Ebola and MERS, have shown that healthcare workers are vulnerable to mental health problems (10–12) and that some consequences can be persistent (11). In the current COVID-19 pandemic, a recent systematic review and meta-analysis showed a high prevalence of depression (31.1%) and posttraumatic stress disorder (PTSD; 31.4%) among caregivers in practice worldwide (13). PTSD is a mental health problem that affects people who are exposed to potentially traumatic events. In particular, healthcare workers are vulnerable to PTSD because they are directly exposed to COVID-19 trauma, including the death of patients due to COVID-19, danger of contamination and the possibility of transmitting SARS-Cov2 to another person (14, 15). However, until now, no studies have investigated symptoms of PTSD for traumas specifically related to COVID-19 in healthcare professionals. Regarding Brazil, a web survey conducted at the beginning of the COVID-19 pandemic showed that living in Brazil was associated with increased odds of depression among essential workers, which can be explained in part by the additional social, structural and political problems in Brazil (16).

Developing strategies to protect mental health, especially in this population, is an important task for governments and health systems around the world (17), especially in countries with great inequalities in income/wealth, such as Brazil (18). An important step is to identify vulnerability and protective factors to prevent mental disorders from progressing (19), which becomes even more relevant and challenging when applied in the context of the COVID-19 pandemic.

Insights about vulnerability and protective factors that impact the mental health of healthcare workers during the COVID-19 pandemic have already been provided by studies investigating many objective aspects, such as years of work, professional level, gender and age (20–25). Here, we focused on aspects about the self-perception of daily professional life in dealing with COVID-19 that are still relatively unknown, such as the perception of stress from being isolated, professional recognition, and altruistic acceptance of risk.

In line with this notion, for example, most studies showing negative associations between social isolation and mental health outcomes during the COVID-19 pandemic have evaluated objective aspects of isolation (e.g., duration of isolation, local structure in which isolation occurs and comparisons of the mental health outcomes of individuals who were isolated from those who were not) (26–29). Furthermore, studies exploring the psychological impact of social isolation in healthcare professionals during COVID-19 remain scarce, and the role of self-perceived level of stress from being isolated from one's family in predicting psychiatric symptoms remains undetermined. It is important to emphasize here that, although subjective feelings of social isolation and the objective state of social isolation frequently co-occur, studies have suggested that they are not equal; both can exert a detrimental effect on health through shared and different pathways (30).

In general, much less attention has been given to factors that could be associated with protection against poor mental health outcomes during an epidemic, including self-perceived professional recognition and altruistic acceptance of risk. According to findings from previous epidemics, professional recognition can be considered a motivating factor for medical teams to continue working in future epidemics (31). In the current COVID-19 pandemic, professional recognition has emerged as a significant protective factor against burnout syndrome (32); however, it is necessary to expand knowledge to other psychiatric conditions, such as PTSD and depression. Altruistic intent to help, a quality frequently found among healthcare workers, was related to a statistically significant decrease in PTSD and depression symptoms during the SARS outbreak among hospital employees in Beijing, China (11, 12). While altruistic intent to help has been shown to be protective against psychiatric symptoms in previous epidemics, no studies have assessed the role of perceived altruistic acceptance of risk in the prediction of depressive and PTSD symptom severity levels during the COVID-19 pandemic.

Identifying vulnerability and protective factors for mental health is a major challenge in psychiatry. Currently, we can apply artificial intelligence, such as machine learning approaches, to find individual predictions that can help to detect mental health vulnerabilities (33–35). Machine learning is a rapidly emerging field that has the potential to identify multivariate patterns in psychometric data that enable the classification of an independent series of individuals (classification model) or the prediction of continuous variables (such as symptoms) at the individual subject level (pattern regression model). In fact, pattern regression models could allow for the investigation of mental health outcomes that represent vulnerability to or protection against the severity of psychiatric symptoms. However, there are still few studies using pattern regression models to predict mental health symptoms based on psychometric data during the COVID-19 pandemic (37–39). Here, we aimed to apply pattern regression models based on psychometric data to predict depression and PTSD symptoms among healthcare workers during the COVID-19 pandemic.

A fundamental insight from the field of statistical learning is that the ability of a model to predict the values of new observations will generally be overestimated based on the fit of the model to a particular dataset [(39); for a review, see (40)]. In the context of machine learning, the term “predict” means that, once the model has learned a relationship between a set of patterns (e.g., multivariate patterns of psychometric data) and labels (e.g., a clinical score), given a new pattern (e.g., psychometric data from a new subject), it can predict its label. Despite being innovative, the advantages of this method include the following: (1) models are not constrained by traditional assumptions, such as a normal distribution of the data or an a priori model; (2) the method can evaluate relationships among many variables at once; and (3) it is particularly helpful for finding patterns in complex datasets (41).

In summary, the present study aimed to apply a machine learning approach (pattern regression model) for the first time to predict depression and PTSD symptoms regarding traumatic events specifically related to the pandemic based on self-perceived (1) level of stress from being physically isolated from one's family; (1) professional recognition before and after the pandemic; and (1) altruistic acceptance of risk during the COVID-19 pandemic among hospital and/or emergency care unit employees. There is an urgent need to identify vulnerability and protective factors for mental health, especially for healthcare workers, to implement targeted prevention and intervention programs to reduce the psychiatric burden affecting healthcare systems worldwide during the COVID-19 pandemic.

## Methods and Materials

### Study Design and Recruitment Procedure

This study was part of a broader project, the PSIcovidA project, aimed at investigating the impact of traumatic events related to the COVID-19 pandemic on professionals working in hospital environments or in emergency care units acting directly or indirectly in the fight against the COVID-19 pandemic in Brazil. PSIcovidA has a cross-sectional data and follow-up survey design. This paper presents cross-sectional that were collected over 3 months between June 12, 2020, and September 19, 2020.

Data were collected by a convenience snowball sampling technique from professionals working in different healthcare contexts or in emergency care units in different states of Brazil. An online survey was developed and sent by WhatsApp Messenger (WhatsApp Inc, Mountain View, CA, USA) and e-mail. An Instagram account and a webpage for the PSIcovidA project were created to advertise the project. Furthermore, the professional associations of all major healthcare worker groups in Brazil were contacted to publish the main project proposal and the link to complete the survey online on their websites and on Instagram. Moreover, interviews in Brazilian media about the study were conducted to invite people who worked in hospitals or emergency units to participate.

Participants were asked to complete a set of validated questionnaires that included sociodemographic questions, as well as questions about professional recognition before and during the pandemic; mental disorder symptoms, including symptoms of depression and PTSD; social isolation from one's family; and altruistic acceptance of risk. At the end of the questionnaires, participants were presented with a list of online psychological support groups.

This study was approved by the Ethics Research Committee of Federal Fluminense University (UFF) and National Research Ethics Commission (CONEP) under process number CAAE 31044420.9.0000.5243, and all of the participants agreed to participate voluntarily in the survey.

### Participants

In total, 1,843 respondents accessed the web survey and completed it. The inclusion criterion was being a hospital and/or emergency health care worker, which generated a sample of 1,399 participants. The exclusion criteria included not having experienced a traumatic event related to the COVID-19 pandemic situation (*n* = 220) or having failed to fully complete the questionnaire battery (*n* = 178). Furthermore, 564 participants who had not experienced some level of isolation, i.e., physical distance from one or more family members, such as children, brothers, husbands or wives, for at least 1 week at the time of the pandemic were also excluded. After the application of these criteria, the final sample consisted of 437 respondents representing all 26 states in Brazil. The majority of our sample consisted of women (*n* = 320, 73.2%), 20-72 years (M = 39.5; SD = 10.8, range: 20-72 years), and a large proportion of the respondents lived in the state of Rio de Janeiro (62%). The sample mirrored the Brazilian population of healthcare workers in terms of gender. Estimates by the National Council of Municipal Health Secretariats (CONASEMS), based on Brazilian Institute of Geography and Statistics (IBGE) data, indicate that women represent 65% of the more than six million professionals working in the public and private health sectors at all levels of care complexity (42). Additional sociodemographic information is presented below (Table 1).

**Table 1:** Sociodemographic and occupational characteristics of the participants.

		***N* (%); Mean (SD)**
**Sociodemographic characteristic**		
**Gender**		
	Female	320 (73.2%)
	Male	117 (27.8%)
**Age**		39.5 (10.8)
**Professional level**		
	Technician	87 (19.9%)
	Superior	350 (80.1%)
**Profession**		
	Medical doctor	173 (39.6%)
	Nurse	72 (16.5%)
	Nurse technician	60 (13.7%)
	Physiotherapist	43 (9.8%)
	Clinical psychologist	27 (6.2%)
	Pharmacist	19 (4.4%)
	Other	43 (9.8%)
**Region**		
	Southeast	321 (73.5%)
	South	34 (7.8%)
	North	18 (4.1%)
	Northeast	57 (13.0%)
	Midwest	7 (1.6%)
**Institution**		
	Public	228 (52.2%)
	Private	86 (19.7%)
	Both	123 (28.1%)
**Presence of mental disorder**		
	No	309 (70.7)
	Yes	128 (29.3)
**Worst trauma Covid**		
	Learning about the death of a close relative or coworker	94 (21.5%)
	Possibly transmitting the COVID-19 virus to another person	90 (20.6%)
	Experiencing the imminent risk of death of a close relative or coworker	72 (16.5%)
	Personally witnessing the death of a patient	67 (15.3%)
	Being infected with COVID-19	48 (11.0%)
	Being exposed to infected patients at high risk for death	47 (10.8%)
	Personally witnessing the death of a close relative or coworker	19 (4.3%)

### Predictive Variables—Psychometric Questions

The pattern regression models included three different psychometric questions assessing vulnerability and protective factors for mental health disorders (PTSD and depression symptoms).

#### Professional Recognition

Respondents were asked to rate their perceived professional recognition before and during the pandemic using a 10-point Likert scale. In particular, they were asked to answer the following question: “In your opinion, from 1 to 10, how much did the general population appreciate healthcare professionals?” (1 = not at all, 10 = too much).

#### Altruistic Acceptance of Risk

The item “Because I wanted to help the COVID-19 patients, I was willing to accept the risks involved” was used as a measure of altruistic acceptance of risk. Respondents were asked to rate this item from 1 (not at all) to 10 (extremely true). This question was adapted from the 10th item of the Perceived Threat Questionnaire developed by Chong et al. during the SARS pandemic (43).

#### Stress Due to Social Isolation

The respondents were asked to rate their level of stress due to being isolated from one or more members of their families for at least 1 week at the time of pandemic using a 10-point rating scale (1 = low, 10 = high).

### Variables to Be Predicted—Psychometric Scales for PTSD Symptoms and Depression Symptoms

#### Posttraumatic Stress Disorder Checklist 5

Posttraumatic stress symptoms were assessed using the PCL-5, which was developed by the National Center for PTSD in accordance with the DSM-5 criteria (44, 45). This scale was translated and adapted to Portuguese by Lima et al. (46). The PCL-5 is a 20-item self-report questionnaire that measures four clusters of symptoms of PTSD: intrusion, avoidance, negative alterations in cognition and mood, and alterations in arousal and reactivity. Each item on the PCL-5 questionnaire is assessed *via* a five-point Likert scale (from 0 = not at all to 4 = extremely). Symptom severity can be calculated by totaling the items for each of the four clusters or totaling all 20 items; in this case, the severity score ranged from zero to 80 points.

The participants were instructed to complete the PCL-5 in relation to their worst traumatic experience related to the COVID-19 pandemic. To assess the worst trauma, we developed a questionnaire composed of seven items that investigated traumatic situations experienced during the COVID-19 pandemic and the level of stress associated with them. These situations included (1) personally witnessing the death of a patient due to COVID-19; (2) personally witnessing the death of a family member or coworker due to COVID-19; (3) learning, through others, about the death of a family member or a coworker due to COVID-19; (4) experiencing the imminent risk of death of a family member or coworker due to COVID-19; (5) being exposed to critically ill patients infected with COVID-19 whose lives were in danger; (6) being infected with COVID-19; and (7) believing or having confirmation that one might have transmitted the virus someone very close (coworker, partner, friend or family). All of these items are in accordance with criteria A for the development of PTSD in the DSM-5. A trauma index question was also used that asked participants to choose their worst experience considering the previous questions and how long ago the event occurred (less or more than 1 month ago). After completing this questionnaire, the participant indicated the worst trauma experienced.

#### Depression

The Patient Health Questionnaire 9 (PHQ-9) is a 9-item self-report questionnaire that assesses symptoms of major depression based on the DSM-IV criteria (47). The nine symptoms are depressed mood, anhedonia, problems with sleep, tiredness or lack of energy, change in appetite or weight, feelings of guilt or worthlessness, problems with concentration, feeling slow or restless and thoughts of suicide. The PHQ-9 score ranges from 0 to 27 points, and each of the 9 questions can be scored from 0 (not at all) to 3 (nearly every day). Here, we used the Brazilian–Portuguese version of the PHQ-9 (48).

The table below shows the means and standard deviations for the psychometric questions and scales (Table 2). Importantly, the level of professional recognition was significantly higher during the pandemic than before the COVID-19 pandemic (*t*-test, *P* < 0.001).

**Table 2:** The means and standard deviations for the psychometric questions and the scales in the considered sample.

**Factor**	**Variable**	**Mean (SD)**
**Protective factors**		
	Professional recognition (before the pandemic)	4.4 (1.9)
	Professional recognition (during the pandemic)	7.2 (2.0)
	Altruistic acceptance of risk	7.1 (2.6)
**Risk factor**		
	Stress due to social isolation	7.6 (2.3)
**Psychiatric symptoms**		
	Model1 (PTSD, PCL-5)	28.6 (17.7)
	Model2 (Depression, PHQ-9)	10.7 (6.8)

### Pattern Regression Analysis

We used pattern regression analysis to predict mental health outcomes (depression or posttraumatic stress symptoms) based on psychometric questions, including: (1) level of stress due to being isolated from one's family; (2) professional recognition before and during the COVID-19 pandemic; and (3) altruistic acceptance of risk before the pandemic. More specifically, we trained two regression models with the goal of predicting posttraumatic stress symptoms (model 1) and depression symptoms (model 2).

Pattern regression analyses were implemented in the Pattern Recognition for Neuroimaging Toolbox (PRoNTo), version 3 (49). The procedure for building pattern regression models consists of two phases: training and testing. During the training phase, the model was trained by providing examples of psychometric questions (i.e., professional recognition before and during the pandemic, altruistic acceptance of risk and stress due to social isolation) and a label (variables to be predicted: posttraumatic or depression symptoms). Once the model “learned” the association between the question scores and the label from the training data (i.e., the model parameters were estimated based on the training data), it could be used to predict the label of a new test example (i.e., scores of PTSD/depression scale). The output of the model is the predicted clinical score obtained during the test phase. The sum of each psychometric question score was included in the model separately: professional recognition before the pandemic (1) and during the pandemic (2), altruistic acceptance of risk (3) and the level of stress due to being isolated from one's family (4).

Linear epsilon-insensitive support vector machine (ε-SVM) regression was applied to predict posttraumatic symptoms and depression symptoms based on psychometric questions. The choice of machine learning algorithm depends on many factors, such as the generalization performance measured on test data and the computational cost of the algorithm. In this study, we applied a non-kernel regression algorithm: the linear ε-SVM. In preliminary investigations, we compared the performance of three different algorithms currently available in PRoNTo: ε-SVM, gaussian process regression (GPR) (50) and kernel ridge regression (KRR) (51). There were no significant differences in performance among the three different approaches. For the sake of brevity, we chose to present results only for ε-SVM. Furthermore, SVM is considered better than most of the other algorithms used because it has better accuracy in its results, especially for smaller samples. Since their introduction in 1992, SVMs have been studied, generalized, and applied to several problems. Furthermore, SVM is relatively stable and memory efficient and has been extensively used for regression models (52–54).

Essentially, ε-SVM performs linear regression in a high-dimensional space using epsilon-insensitive loss, also known as L1 loss. In ε-SVM, the user must set two hyperparameters, ε and C, either manually or using a cross-validation scheme. The hyperparameter ε defines a margin of width ε around the regression line, setting a margin of “tolerance,” where any data point that falls within it carries no penalty. The C hyperparameter, in contrast, controls how strongly data points beyond the epsilon-insensitive margin are penalized. In essence, ε sets a margin outside of which data points are penalized, and C defines the penalty itself. The idea is similar to the concept of a “soft margin” in SVM classification (55). Both hyperparameters were automatically optimized in PRoNTo using a two-fold nested cross-validation procedure, with the same cross-validation scheme for the internal and external loops.

In this case, there are two loops in the cross-validation scheme. The inner loop is used for parameter optimization, and the outer loop is used for assessing the model's performance. More specifically, the data are divided into training and testing sets according to the cross-validation scheme selected (outer loop). For each fold of the outer loop, the training set is further divided into training and testing sets according to the cross-validation scheme selected (inner/nested loop). The inner loop is used to train and test the model with each value of the hyperparameter specified by the user. The parameter leading to the highest performance in the inner/nested loop (according to the mean squared error) is then used in the outer loop. For each fold of the outer loop, the model is trained using the “optimal” value of the hyperparameter and tested on the data that were omitted (and which were not used for parameter optimization). PRoNTo allows for the automatic optimization of more than one parameter, entered as a cell array of values that is then transformed into a grid. The parameters used were values of 0.01, 0.1, 1, 10, 100, and 1,000.

**Figure 1:**
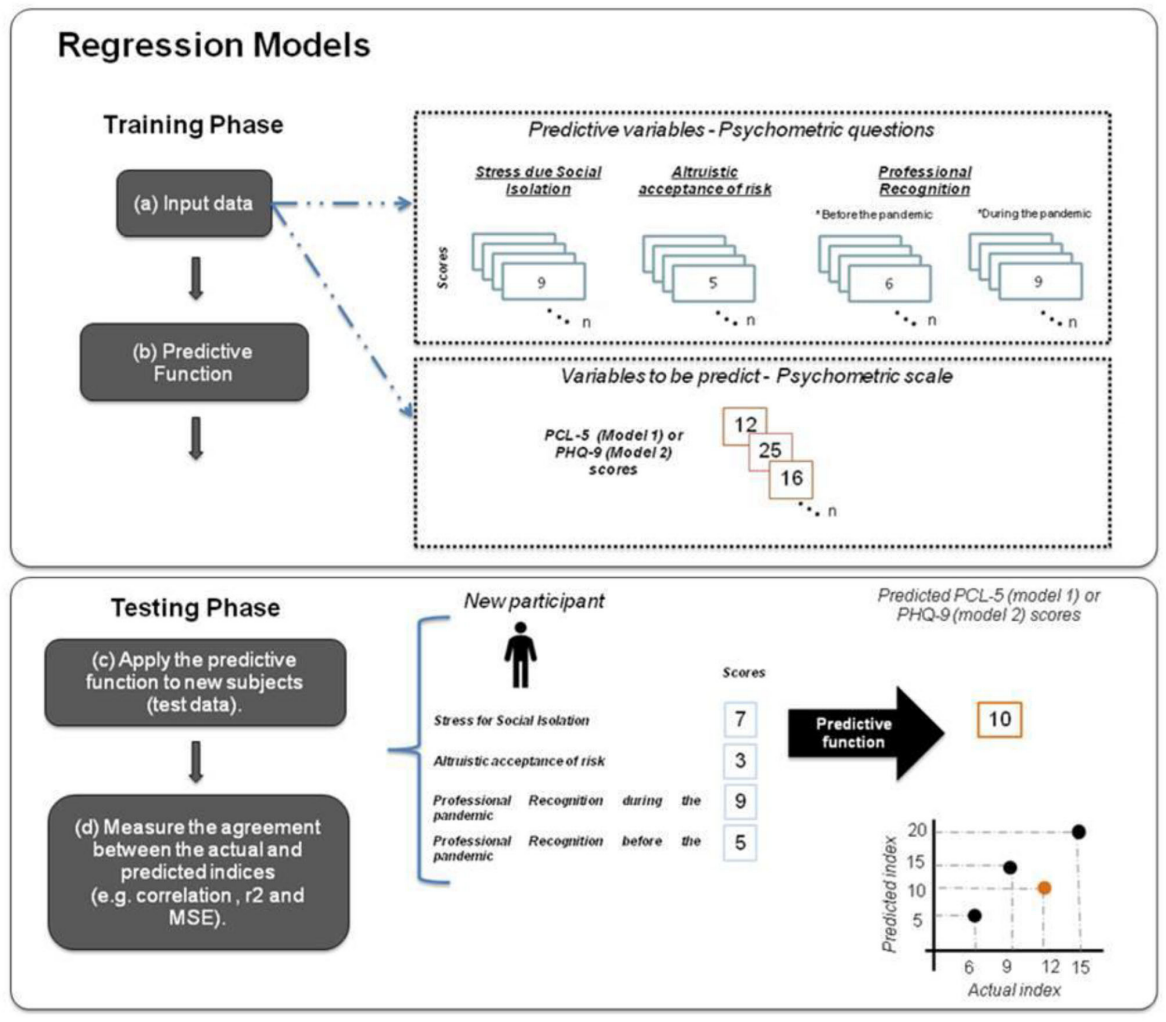
Regression models: (a) The training data for the ε-SVM regression model consists of examples that pair the psychometric factors (stress due to social isolation, altruistic acceptance of risk and professional recognition before and during the pandemic) of each subject and the corresponding clinical score (PCL-5 or PHQ-9). (b) During the training, the ε-SVM model learns the contribution of each psychometric question for the predictive function. (c) During the testing phase, given the psychometric questions of a test subject, the ε-SVM model predicts its corresponding clinical score. (d) The model performance is evaluated using three metrics that measure the agreement between the predicted and actual clinical scores: Pearson's correlation coefficient (r), coefficient of determination (r^2^) and normalized mean squared error (NMSE).

To evaluate the ε-SVM performance we used two different cross-validation strategies (a two-fold cross-validation and a five-fold cross-validation) to demonstrate that the results were not dependent on a specific cross-validation scheme. We choose two and five-fold cross validation, as these numbers of splits seemed reasonable considering our sample size. A two-fold cross-validation procedure means that the sample was divided in two, with half of the sample used for training and half used for testing in the first fold and with the half of the sample that was used for testing then being used for training and vice versa in the second fold (see Figure 1). The five-fold cross validation involves dividing the data into five disjoint sets. Data from each set is left out once for test and data from the remaining four sets are used to train the model. This procedure is then repeated five times, so that each set is left out once. In both cases, the performance of the model is computed based on the concatenation of the predictions across folds, as implemented in PRoNTo.

Regarding potential confounders, being female, being younger and reporting a current mental health diagnosis have previously been associated with depression among essential workers in Brazil (16). However, removing confounders associated with the variable to be predicted (i.e., the labels) is not recommended because this adjustment is likely to remove not only the variability in the data due to confounding factors but also the variability in the data associated with the labels (56, 57). To address this limitation, we balanced the proportion of data from potential confounders across the different folds. There was no difference in the distribution of the sample regarding the presence of mental disorders diagnosed before the pandemic, gender, age or the scores on the questions and PTSD/depression symptoms for both cross-validation strategies (see Supplementary Tables 1, 2).

### Performance of the Model

To determine the performance of the regression model, three metrics were used to measure the agreement between the predicted and actual PTSD/depression symptoms: Pearson's correlation coefficient (r), the coefficient of determination (r^2^) and the normalized mean squared error (NMSE). The correlation coefficient (r) describes the strength of a linear relationship between two variables. A small correlation is an indication of poor predictive performance. The coefficient of determination (r^2^) can be interpreted as the proportion of variance explained by the regression. The NMSE is the mean of the squared differences between the predicted and true scores; it represents the mean error between the predicted and actual scores and is commonly used to evaluate the performance of predictive models. The MSE was normalized by dividing the MSE by the variance in the target values.

The significance of the regression performance measures was determined using permutation tests, i.e., the same cross-validation procedure described above was performed 1,000 times with the labels permuted across the participants. The *P*-value was calculated by counting how many times the absolute value of the metric with the permuted labels was equal to or greater (less for MSE) than the absolute value of the metric obtained with the correct labels and dividing by 1,000. The results were considered significant when the model performed equal to or better than the model without shuffling the labels at most 5% of the time across 1,000 permutations (58).

### Model Interpretation

The weights represent the contribution of each psychometric question to the linear predictive function and can be explicitly computed and plotted for interpretation and discussion. As previously discussed in the literature (58), the weight map of linear machine learning models cannot be thresholded to make specific inferences as in classical (univariate) techniques. Since each cross-validation fold yields a different weight vector, the final psychometric weight is the average across the folds divided by its Euclidean norm. For the sake of brevity, we illustrate only the two-fold cross- validation in the manuscript.

## Results

### Pattern Regression Model

**Figure 2:**
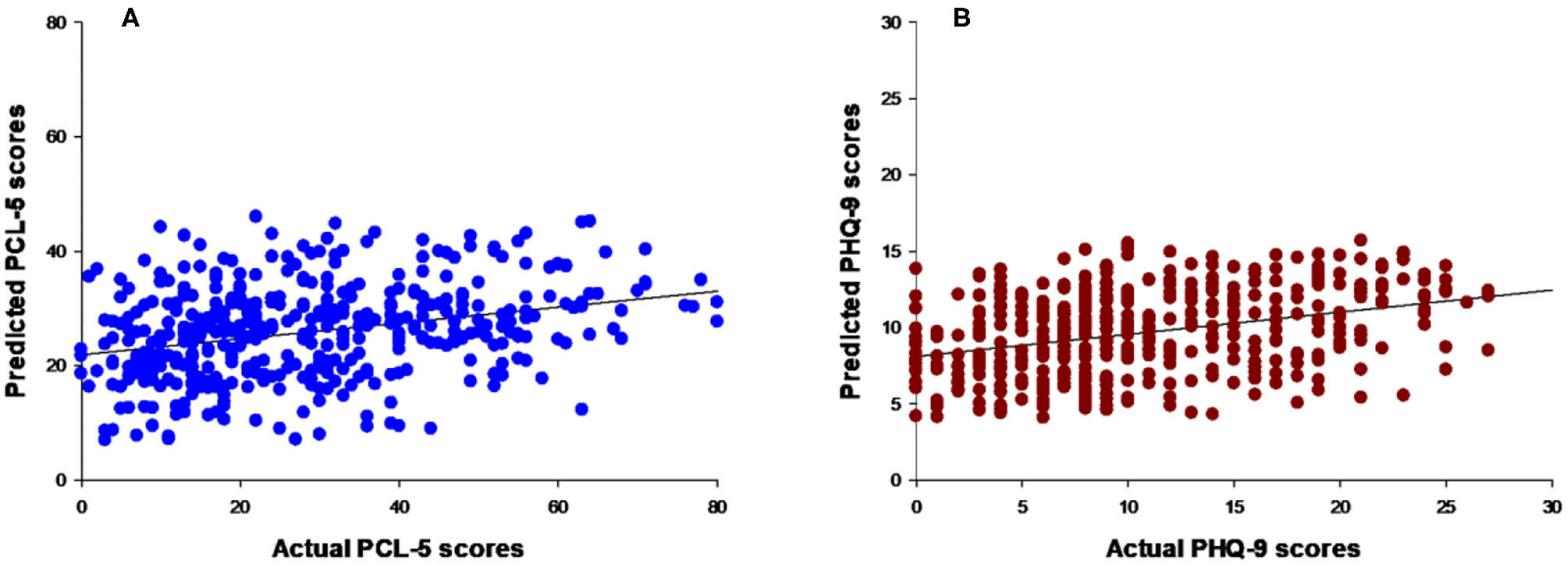
Scatter plots of actual vs. predicted values applying a two-fold cross-validation scheme for the PTSD symptoms model and for the depression model. **(A)** Scatter plot between the actual and predicted PCL-5 scores (PTSD symptoms model). **(B)** Scatter plot between the actual and predicted PHQ-9 scores (depression model).

After correction for multiple comparisons (since four different models were tested, the significance threshold was 0.05/4 = 0.0125), the ε-SVM regression models significantly predicted PTSD and depression symptoms from the psychometric questions that potentially represented vulnerability/protective factors for mental disorders. For PTSD, the performance of the regression model is presented in Table 3 [twofold: r = 0.35 (*P*-value = 0.001), r^2^ = 0.12 (*P*-value = 0.001) and NMSE = 0.96 (*P*-value = 0.001), and five-fold: r = 0.34 (*P*-value = 0.001), r^2^ = 0.12 (*P*-value = 0.001) and NMSE = 0.90 (*P*-value = 0.001)]. Figure 2A shows a scatter plot depicting the predicted vs. actual PTSD symptoms for the two-fold cross-validation. Similar results were obtained for depression symptoms [two-fold: r = 0.36 (*P*-value = 0.001), r^2^ = 0.13 (*P*-value = 0.001) and NMSE = 0.90 (*P*-value = 0.001), and five-fold: r = 0.38 (*P*-value = 0.001), r^2^ = 0.15 (*P*-value = 0.001) and NMSE = 0.86 (*P-*value = 0.001)], indicating that our models significantly decoded both PTSD and depression symptoms from psychometric questions (Table 3; Figure 2B). There were no significant differences in performance among the different kernel regression approaches and non-kernel approaches (see Supplementary Tables 1, 3 to consistency of results).

**Table 3:** Measurements of agreement between the actual and decoded scores based on scores of professional recognition, altruistic acceptance of risk and stress level due to social isolation.

**Models**	**Cross-validation schemes**		**Measures of agreement**	
		**r (*P*-value)**	**r^**2**^ (*P*-value)**	**NMSE (*P-*value)**
PTSD	“Two-fold”	0.35 (0.001)	0.12 (0.001)	0.96 (0.001)
	“Five-fold”	0.34 (0.001)	0.12 (0.001)	0.90 (0.001)
Depression	“Two-fold”	0.36 (0.001)	0.13 (0.001)	0.90 (0.001)
	“Five-fold”	0.38 (0.001)	0.15 (0.001)	0.86 (0.001)

* *For reference: corrected p-value = 0.0125*.

### Contributions of Psychometric Questions to the Regression Model

**Figure 3:**
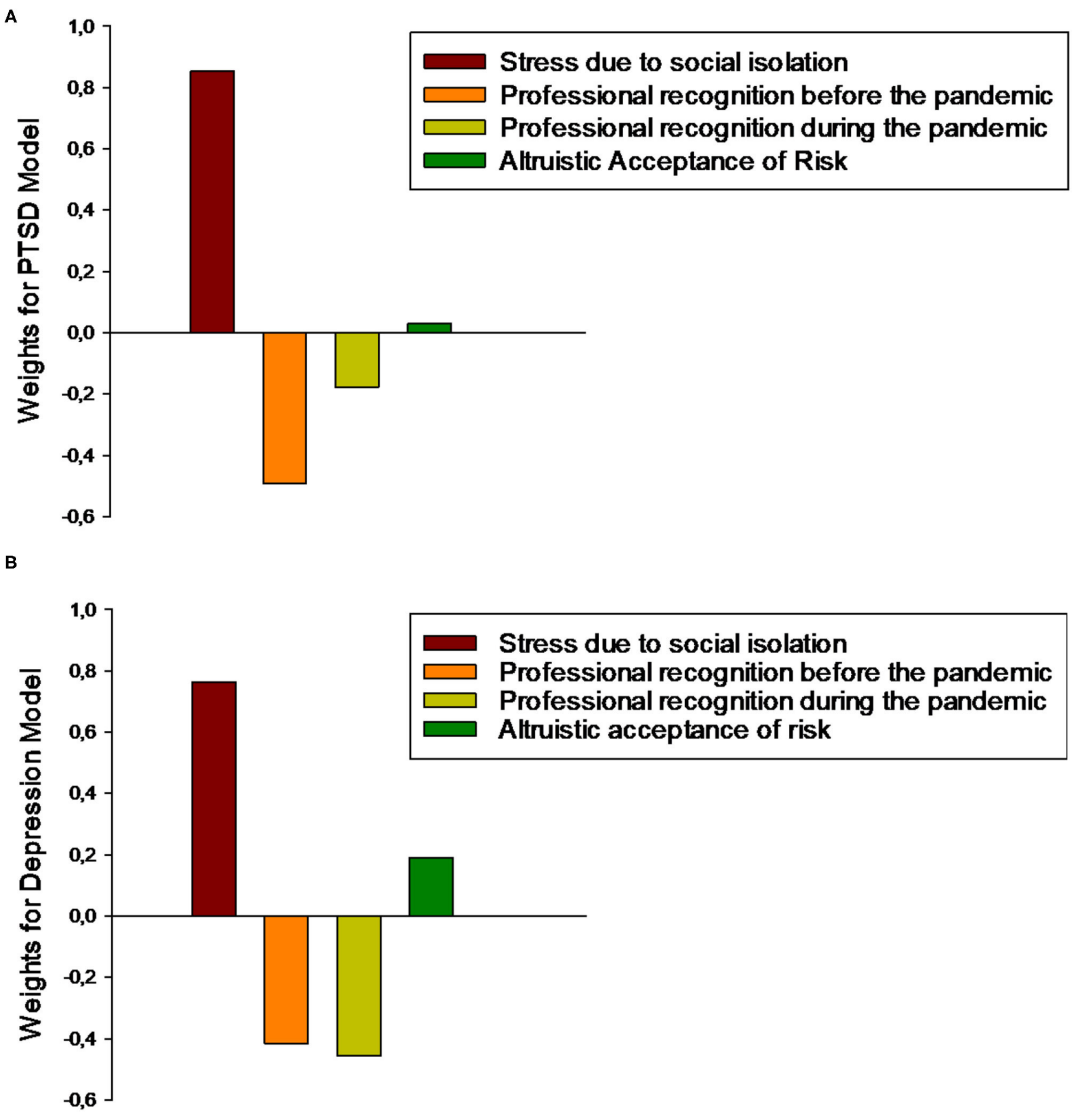
**(A)** Plot showing the values of the weights for each scale for the prediction of PTSD symptoms. **(B)** Plot showing the values of the weights for each scale for the prediction of depression symptoms.

For the sake of brevity, we display the weight maps only for the model based on the two-fold cross-validation scheme in the main manuscript. The relative contribution of each psychometric question to the ε-SVM for both models is shown in Figure 3. The weight of each psychometric question corresponds to its contribution to the model's prediction. Notably, for the PTSD model, the psychometric questions with the greatest contributions were the level of stress due to social isolation (0.85) and professional recognition, mainly before the pandemic (before = −0.49 and during = −0.18), and the psychometric question making the smallest contribution was altruistic acceptance of risk (0.03). Similar results were obtained for the depression model, in which the level of stress due to social isolation (0.85) and professional recognition (before = −0.49 and during = −0.18) made the greatest contributions and the psychometric question making the smallest contribution was altruistic acceptance of risk (0.03). Interestingly, professional recognition had negative predictive value, indicating inverse relationships with PTSD and depression.

## Discussion

There were many new cases and deaths during the data collection (3), revealing the pandemic's impact in Brazil. Consequently, intense demand was imposed on healthcare workers, leading to greater pressure on mental health services in Brazil. The main goal of the present study was to apply machine learning, particularly pattern regression analysis, to determine the impact of the self-perceived level of stress due to social isolation, professional recognition and altruistic acceptance of risk on the mental health outcomes (depression and PTSD symptoms) of employees working in hospitals and/or emergency care services during the COVID-19 pandemic. The results confirmed that ε-SVM models were able to predict PTSD symptoms (PCL-5 scores) and depression symptoms (PHQ-9 scores). For both models, the self-perceived level of stress due to social isolation and professional recognition were the variables making the greatest contributions to the predictive function. Interestingly, professional recognition had negative predictive value, indicating an inverse relationship with posttraumatic and depression symptoms. These findings suggest that hospital workers who have higher levels of self-perceived levels of stress due to being isolated from one or more members of their families could be more vulnerable to experiencing psychiatric symptoms. Furthermore, our results indicate that professional recognition might be an important protective factor for the mental health of hospitals and emergency care workers. Finally, our results are promising since they suggest that machine learning algorithms could provide significant models for predicting mental health symptoms from psychometric data. To our knowledge, this study is the first showing that the perception of stress from being isolated and professional recognition are very important factors to be considered for Brazilian healthcare workers' mental health conditions. Such knowledge is relevant for devising preventive measures and care actions at occupational and institutional levels, considering the importance of the current context.

An important strength of the present study was the assessment of traumatic events specifically related to COVID-19 pandemics. The participants answered questions that investigated potentially traumatic situations experienced by healthcare workers since the COVID-19 outbreak. The traumatic experience related to COVID-19 most frequently reported was “learning about the death of a close relative or coworker, due to COVID-19” followed by “possibly transmitting the COVID-19 virus to another person.” This finding is in agreement with previous studies about trauma prevalence before the pandemic since trauma related to death of a beloved one has been reported to be the most frequent trauma (59).

Throughout this pandemic healthcare workers have had to self-isolate from their own families mainly due to fear of transmitting the virus to their loved ones. However, since humans are highly social and cooperative animals, the response to the threat of infection by COVID-19 causes the desire for physical contact, especially in relation to loved ones, such as family members (60). In fact, adequate social contact is critical for mental health (61). For these reasons, humans struggle when forced to live in isolation, and most of us find social deprivation stressful. In fact, our data indicate that the loss of this type of social contact can impact the mental health of healthcare workers. In our pattern regression models, the level of stress due to isolation from one's family for at least one week was a relevant factor for PTSD and depression symptoms. This finding is supported by the existing literature on previous epidemics and the current COVID-19 pandemic that has reported negative associations between quarantine/social isolation and mental health outcomes in these professionals (11, 12, 62, 63). Along the same line, a recent meta-analysis focusing on objective measures of isolation reported that individuals experiencing isolation or quarantine were at increased risk for adverse mental health outcomes, particularly after a duration of 1 week or longer (29). To the best of our knowledge, our study is the first to show that the self-perceived level of *stress* due to being isolated from one's family members is a significant and important factor for the severity of psychiatric symptoms in hospital and emergency care workers during the COVID-19 pandemic. One possible explanation for why self-perception of stress leads to psychiatric symptoms came from a study showing that social isolation (self-perception of loneliness during COVID-19) both mediates and moderates the indirect effect of COVID-19 worries on posttraumatic stress symptoms (PTSS) related to COVID-19 among individuals who have not yet been infected with COVID-19 (64). Further studies should investigate the relationship among COVID-19-related worries, feeling of loneliness and the self-perceived level of stress due to being isolated in predicting PTSD and depression symptoms in healthcare workers.

Conversely, we found that self-perceived professional recognition before and during the pandemic had negative predictive value, indicating an inverse relationship with PTSD and depression symptoms. Importantly, the level of perceived professional recognition was higher during the COVID-19 pandemic than before it. Here, professional recognition refers to the recognition of a person's work by the general population and reflects the following factors: (1) the esteem support factor, which is a type of social support that reassures a person about his or her skills (65); and (2) the construction of the social esteem factor, which is a sense of the recognition of a person's achievements and contributions at work (66). In fact, both factors are negatively associated with negative mental health problems, including burnout symptoms (67–69). We believe that one of the pathways by which professional recognition might protect against the severity of posttraumatic and depression symptoms is enhancing social support and self-esteem among these professionals. Furthermore, professional recognition has been shown to enhance self-determination (70) and work satisfaction (71). In the current pandemic, the findings regarding professional recognition have shown that the recognition of their work and efforts by hospital management could be motivating factors for medical staff to continue working effectively (72). Our findings are in line with Barello's (32) results and extend prior findings to other psychiatric conditions, showing that professional recognition might be considered a relevant protective factor for the severity of posttraumatic and depression symptoms in healthcare workers during the COVID-19 pandemic.

Finally, the psychometric question with the lowest contribution to the model's predictive function was altruistic acceptance of risk, a quality frequently found among healthcare workers (73, 74). One possible explanation for our findings is that the role of altruistic acceptance of risk as a buffer against psychiatric symptoms is inconsistent. Some studies have found that altruistic acceptance of risk was negatively related to psychiatric symptoms in healthcare workers following an epidemic outbreak (11, 12). These findings indicate that altruistic acceptance of risk might have protected some hospital employees against negative psychological outcomes following the epidemic outbreak. In contrast, other studies have reported that altruistic acceptance of risk was not related to psychiatric symptoms among hospital employees following epidemic outbreaks and the general population (75, 76). However, as emphasized above, these findings should be interpreted with caution since all predictive questions contributed to the final prediction.

Machine learning tools, specifically pattern regression, have been successfully applied with many types of data, such as neuroimaging data (33–35, 77, 78). However, their use has been less investigated in studies using psychometric data (79). In the context of the COVID-19 pandemic, few studies have applied pattern regression based on psychometric data to predict continuous variables among general samples (36, 37) and university student samples (38). Our results are promising since they suggest that machine learning algorithms could provide significant models for predicting mental health symptoms from psychometric data.

There were also some limitations to the present study. First, the sample was not representative of the entire Brazilian healthcare worker population since the data were obtained by a convenience snowball sampling technique via a link sent by WhatsApp and e-mail. While online recruitment guarantees large samples, it does not guarantee sample representativeness. To reduce this limitation, we contacted all major healthcare worker groups in Brazil to publish the main project proposal and the link to complete the survey online on their websites and on Instagram. Additionally, there might have been selection bias. For example, the southeastern region (73.5%) was overrepresented, and we cannot ignore that our results could have been driven by the highest socioeconomic region in Brazil. For example, death and comorbid disease were more common among Brazilians from the North region than among those from the Central-South regions (80). Furthermore, the worst public health and social scenarios were present in the northern regions of Brazil (81). These regions were underrepresented in our sample (17.16%), and it seems important to emphasize that this scenario seen in the Northeast/North regions could worsen the consequences of COVID-19 on the mental health of health care workers. Second, the use of self-report measures did not enable us to verify the reliability of the responses or to ensure that participants correctly understood the questions. Furthermore, to minimize that we did not apply any objective quality control to ensure that the online survey results were credible, we offered anonymity on self-administered questionnaires to reduce social desirability bias, and we also attempted to develop a more concise questionnaire to avoid tiredness. Future research should seek to compare the present study data with those collected using other methods (e.g., semistructured interviews, qualitative approaches, etc.). Another important limitation is that, since removing confounders associated with the variable to be predicted is not recommended, we cannot exclude our results perhaps being influenced by some objective aspects that impact the mental health of healthcare workers during the COVID-19 pandemic, such as working years, professional level, and working on the front line of the hospital. Furthermore, although we used two cross-validation schemes (two-fold (or half split) cross-validation and five-fold cross-validation), predictive models should ideally be further validated with a truly independent sample. Finally, with regard to hyperparameter optimizations, it should be noted that a more automatic fine-tuning technology such as Bayesian optimization may be a good option in the future (82, 83).

The COVID-19 pandemic is still unfolding, and it is likely that the virus and its consequences will impact the health system for some time to come. Identifying vulnerability and protective factors to prevent mental disorders from progressing in healthcare professionals is necessary to promote prevention strategies and to counteract stressors and challenges during this outbreak. Our study findings draw our attention to the predictive role of the level of self-perceived stress due to social isolation in the severity of PTSD and depression symptoms. Furthermore, our findings emphasize the protective role of professional recognition in posttraumatic and depression symptomatology. We suggest here that self-perceived stress due to social distancing and self-perceived professional recognition might also represent important vulnerabilities to be assessed in clinical interviews. Bringing these aspects into the clinical assessment could help clinicians to estimate the risk of worsening PTSD or depression in these professionals. Based on our findings, appropriate action to monitor and reduce the level of stress due to social isolation from family among these groups of individuals working on the frontline of the pandemic should be undertaken immediately. Measuring the degree of self-perceived stress due to social isolation is an important addition to mental health assessments during the COVID-19 pandemic. Stress and social isolation can impact health and immune function, for example, decreasing inflammatory control and viral immunity (84–86). Therefore, reducing the level of stress due to social isolation is essential during a time when individuals require strong immune function to fight off a novel virus. For instance, one possible action to mitigate the consequences of the level of stress due to being isolated is to encourage vulnerable individuals to remain in regular contact with family and friends through video chats, phone calls and online groups. The use of video-embedded digital communication is likely to gain importance. The visual component of interpersonal encounters appears to play a key role in creating a more satisfying experience of digital social media (87). Strategies to foster a sense of belonging among healthcare workers should be encouraged. For example, being connected with or reading stories from people who are also isolated from their families can promote identification and, consequently, emotional comfort. In fact, sense of belonging is a key buffering factor against feelings of stress among healthcare workers during the COVID-19 pandemic (88). Work environments that facilitate these basic psychological needs to feel connected to others and to have a sense of belonging prompt positive psychological outcomes, such as enhanced performance and greater psychological well-being (89–91).

This mobilization now will allow the public health system to apply the knowledge gained to any future periods of increased infection and lockdowns, which will be particularly crucial for healthcare workers and to future pandemics. Our findings strongly suggest that positive recognition experiences can be fostered by hospital management to buffer against negative effects on mental health among healthcare workers for example, creating a program to improve the self-perception of being recognized by the institution can be a very effective way to protect the mental health of these professionals. Within this framework of thought, practical issues, such as salary valorization and improvement of work environment conditions, which include work healthcare centers, readjustment of work environments, humanized leaderships, suppliers of consumables, materials, and individual protection equipment, could contribute to the perception of professional recognition (92). Additionally, outreach in the media and government could encourage the population to recognize issues concerning professional significance. In addition, technical support (room for communication, support staffed by mental health professionals, periodic monitoring of mental health, space to therapeutic interventions, psychoeducation about symptoms for early identification of mental disorders, meditation and mindfulness techniques, physical exercise incentives, etc.) could demonstrate that professionals' mental health is appreciated by the organization (93). Finally, we hope that the COVID-19 pandemic will prompt the recognition of the contributions of all healthcare workers with appropriate protection and compensation, such as wage appreciation.

In summary, this study showed that a machine learning approach (pattern regression model) was able to predict mental health outcomes and PTSD and depression symptoms in healthcare workers based on the self-perceived level of stress due to isolation and professional recognition. These results add to the literature indicating the importance of considering how each healthcare worker perceives the stress of isolation and professional recognition, in addition to more objective factors, such as years of work, professional level, gender, and age. We suggest that it is a fundamental aspect of implementing targeted clinical evaluations and intervention programs within institutions to reduce the psychiatric burden on health systems worldwide during the COVID-19 pandemic and even future pandemics.

## Data Availability Statement

The raw data supporting the conclusions of this article will be made available by the authors, without undue reservation.

## Ethics Statement

The studies involving human participants were reviewed and approved by Research Ethics Committee of the Faculty of Medicine, UFF. The patients/participants provided their written informed consent to participate in this study.

## Author Contributions

LP analyzed and interpreted the data, wrote the manuscript, and contributed to the data organization. CG contributed to the data organization, data interpretation, and revision of the manuscript. RG, FE, IM, KT, MM, EV, and ID interpreted data and revised the manuscript. MP contributed to writing and revising the manuscript and the data interpretation. LO analyzed and interpreted the data and wrote and revised the manuscript. All of the authors read and approved the final manuscript.

## Funding

This work (data collection, analysis and writing) was supported in part by federal and state Brazilian research agencies (CNPq and FAPERJ). Scholarships were awarded by the federal Brazilian research agency CAPES 614 001, CAPES/PRINT.

## Conflict of Interest

The authors declare that the research was conducted in the absence of any commercial or financial relationships that could be construed as a potential conflict of interest.

## Publisher's Note

All claims expressed in this article are solely those of the authors and do not necessarily represent those of their affiliated organizations, or those of the publisher, the editors and the reviewers. Any product that may be evaluated in this article, or claim that may be made by its manufacturer, is not guaranteed or endorsed by the publisher.
