# Supplementary material for: Vulnerability and Protective Factors for PTSD and Depression Symptoms Among Healthcare Workers During COVID-19: A Machine Learning Approach
Source: Front Psychiatry. 2022 Jan 12;12:752870. doi: 10.3389/fpsyt.2021.752870 (PMC8790177; doi:10.3389/fpsyt.2021.752870)
Supplement: Supplementary file 1 [file Data_Sheet_1.docx]

**Table S-1:** The mean and standard deviation obtained for age and all assessment variables in fold 1 and fold 2. t-tests comparing the two folds are also shown.

| Variables | Fold 1 | Fold 2 |  |  |
| --- | --- | --- | --- | --- |
|  | (Mean) | (Mean) | t-test (*P* value) | χ2 (*P* value) |
| Age | 40.26 | 38.66 | 0.12 | - |
| PTSD | 27.80 | 29.35 | 0.36 | - |
| Depression | 10.55 | 10.79 | 0.70 |  |
| Stress due to social isolation | 7.52 | 7.73 | 0.35 | - |
| Professional recognition before the pandemic | 4.37 | 4.38 | 0.95 | - |
| Professional recognition during the pandemic | 7.26 | 7.20 | 0.77 | - |
| Altruistic acceptance of risk | 7.17 | 7.11 | 0.79 | - |
| Gender | - | - | - | 0.89 |
| Current mental health disorder | - | - | - | 0.98 |

**Table S-2: Machine tests for regression models**

| Measure | Machine | r | r^2^ | NMSE |
| --- | --- | --- | --- | --- |
| PTSD | KRR | 0.36 (0.001) | 0.13 (0.001) | 0.87 (0.001) |
|  | GPR | 0.35 (0.001) | 0.13 (0.001) | 0.87 (0.001) |
| Depression | KRR | 0.37 (0.001) | 0.14 (0.001) | 0.86 (0.001) |
|  | GPR | 0.37 (0.001) | 0.14 (0.001) | 0.86 (0.001) |

Note: The *P* value was obtained by a permutation test (1000 permutations). *KRR: kernel ridge regression; GPR: Gaussian process regression*

**Table S-3: Results of each fold in two-fold and five-fold cross-validations for predicting depression and PTSD symptoms.**

| **Depression** | **r** | **R2** | **NMSE** | **PTSD** | **r** | **R2** | **NMSE** |
| --- | --- | --- | --- | --- | --- | --- | --- |
| **Average Twofold** | **0.36** | **0.13** | **0.90** | **Average**  **Twofold** | **0.35** | **0.12** | **0.96** |
| Fold1 | 0.34 | 0.11 | 0.92 | Fold1 | 0.38 | 0.15 | 0.92 |
| Fold2 | 0.38 | 0.15 | 0.87 | Fold2 | 0.31 | 0.10 | 0.99 |
| **Average**  **Fivefold** | **0.38** | **0.13** | **0.86** | **Average**  **Fivefold** | **0.34** | **0.12** | **0.90** |
| Fold1 | 0.42 | 0.18 | 0.87 | Fold1 | 0.28 | 0.08 | 0.98 |
| Fold2 | 0.45 | 0.20 | 0.80 | Fold2 | 0.42 | 0.17 | 0.83 |
| Fold3 | 0.35 | 0.12 | 0.87 | Fold3 | 0.31 | 0.10 | 0.89 |
| Fold 4 | 0.39 | 0.15 | 0.84 | Fold 4 | 0.38 | 0.15 | 0.86 |
| Fold 5 | 0.30 | 0.09 | 0.93 | Fold 5 | 0.32 | 0.10 | 0.95 |
